# Prognostic significance of preoperative prognostic immune and nutritional index in patients with stage I–III colorectal cancer

**DOI:** 10.1186/s12885-022-10405-w

**Published:** 2022-12-16

**Authors:** Hailun Xie, Lishuang Wei, Mingxiang Liu, Yanren Liang, Guanghui Yuan, Shunhui Gao, Qiwen Wang, Xin Lin, Shuangyi Tang, Jialiang Gan

**Affiliations:** 1 Department of Colorectal and Anal Surgery, The First Affiliated Hospital of Guangxi Medical University, 6 Shuangyong Road, Nanning, Guangxi 530021 P.R. China; 2 Guangxi Key Laboratory of Enhanced Recovery After Surgery for Gastrointestinal Cancer, Nanning, Guangxi P.R. China; 3 Department of Geriatric Respiratory Disease Ward, the First Affiliated Hospital, Guangxi Medical University, Nanning, Guangxi P.R. China; 4 Grade 2018, Department of Clinical Medicine, Guangxi Medical University, Nanning, Guangxi P.R. China; 5 Department of Pharmacy, The First Affiliated Hospital of Guangxi Medical University, 6 Shuangyong Road, Guangxi 530021 Nanning, P.R. China

**Keywords:** Colorectal cancer, Overall survival, Disease-free survival, Nutrition, Inflammation

## Abstract

**Background:**

To explore the value of preoperative prognostic immune and nutritional index (PINI) in predicting postoperative complications and long-term outcomes in patients with stage I–III colorectal cancer (CRC).

**Methods:**

Restricted cubic splines were used to assess the relationship between PINI and survival in patients with CRC. The Kaplan–Meier method and log-rank test were used to plot the survival curves. The Cox proportional hazards model was used to evaluate independent prognostic predictors in patients with CRC. A logistic regression analysis was performed to identify independent predictors of postoperative complications. The least absolute shrinkage and selection operator (LASSO) logistic regression algorithm was used for feature screening.

**Results:**

An evident positive dose–response relationship between PINI and survival in patients with CRC was identified. Compared with patients with a high PINI, those with a low PINI had worse disease-free survival (DFS) (47.9% vs. 66.9%, *p* < 0.001) and overall survival (OS) (49.7% vs. 70.2%, *p* < 0.001). The Cox proportional hazards model revealed that PINI was independently associated with DFS (hazard ratio [HR], 0.823; 95% confidence interval [CI], 0.754–0.898; *p* < 0.001) and OS (HR, 0.833; 95% CI, 0.761–0.912; *p* < 0.001) in patients with CRC. In the logistic regression analysis, PINI was an independent factor affecting postoperative complications in patients with CRC (odds ratio, 0.710; 95%CI: 0.610–0.810, *p* < 0.001). The LASSO logistic regression algorithm was used to screen for effective prognostic variables. Finally, we constructed PINI-based nomograms to predict postoperative 1–5-year PFS, and OS in patients with CRC.

**Conclusion:**

PINI is an effective biomarker for predicting postoperative complications, DFS, and OS in patients with stage I–III CRC.

**Supplementary Information:**

The online version contains supplementary material available at 10.1186/s12885-022-10405-w.

## Background

Colorectal cancer (CRC) is one of the most severe malignancies threatening human health and is considered the second leading cause of cancer-related deaths worldwide [1]. In China, CRC has become the second most common malignancy after lung cancer, and its mortality rate is also at the forefront [2]. Pathological stage is currently the most widely used prognostic assessment tool for CRC; however, it is complicated due to its heavy reliance on histopathological specimen evaluation. Pathological stage only assesses tumor characteristics and ignores host-related factors, such as systemic inflammation, and even patients with the same pathological stage can have very different survival outcomes. Survival cannot be fully explained with pathological stage or currently established prognostic factors [3, 4]. Therefore, the potential prognostic biomarkers independent of pathological stage need to be identified in order to effectively predict the prognosis of patients with CRC.

Systemic inflammation mediated by cytokines and immune cells is reportedly related to the occurrence and development of malignancies [5, 6]. Systemic inflammation plays a role in the induction, promotion, transformation, invasion, and metastasis of malignancies. Systemic inflammation can be reflected by serum biomarkers, such as leukocytes, neutrophils, and monocytes, which are constantly being developed for prognostic studies because of their simplicity and convenience [7, 8]. Malnutrition is also an important factor that affects the prognosis of patients with CRC as it is associated with an increased risk of complications and mortality in such patients [9, 10]. Moreover, malnutrition can prolong the length of hospital stay and increase the cost of hospitalization [11]. It also reduces chemotherapy response and leads to chemotherapy-related adverse events [12]. Recently, Jung et al. developed a novel prognostic indicator reflecting systemic inflammation and nutrition, named the prognostic immune and nutritional index (PINI), which was calculated from serum albumin levels and monocyte count [13]. Serum albumin is an important biomarker of nutritional status and is also considered an important inflammatory protein reflecting systemic inflammation [14]. Meanwhile, monocytes, a major inflammatory component of tumors, play a crucial role in tumor development and metastasis by promoting angiogenesis and invasion [15, 16]. Both are effective biomarkers for predicting the prognosis of patients [17, 18]. Therefore, PINI, which combines the advantages of serum albumin and monocytes, may be a promising biomarker for predicting the prognosis of patients with CRC.

PINI has been reported as a prognostic indicator for colon cancer in the Korean population; however, its suitability for the Chinese population remains unclear. Therefore, this study aimed to explore the value of PINI in predicting postoperative complications and long-term outcomes in Chinese patients with stage I–III CRC.

## Methods

### Study population

This study consecutively enrolled patients with stage I–III CRC who received surgery at the Colorectal and Anal Surgery Department of the First Affiliated Hospital of Guangxi Medical University between January 2012 and December 2016. The inclusion criteria were as follows: the pathologically confirmed primary pathological site was the colon or rectum; did not receive preoperative neoadjuvant chemoradiotherapy; and underwent primary tumor resection. The exclusion criteria were as follows: multiple primary malignancies; agranulocytosis, severe infection, or severe immune diseases; and missing general and clinicopathological data.

### Data collection

Clinicopathological data of the enrolled patients were reviewed and collected from the hospital’s medical record system. The general information included sex, age, height, weight, and body mass index (BMI), Information on comorbidities, including a history of hypertension and diabetes, was also collected. Fasting venous blood of the patients was measured within 1 week preoperatively to obtain serological data, including monocyte count, serum albumin level, and serum carcinoembryonic antigen (CEA) level. Pathological features included T stage, N stage, tumor–node–metastasis (TNM) stage, perineural invasion, vascular invasion, pathological type, differentiation, tumor location, and tumor size. All pathological features were obtained by evaluation of resected tissue specimens by professional pathologists, including surgical approach, operative time, intraoperative blood loss, postoperative radiotherapy, and postoperative chemotherapy. According to a previous study [13], PINI was defined as [albumin (g/dL) × 0.9] – [monocyte (mm^3^) × 0.0007]; Neutrophil to lymphocyte ratio (NLR) was defined as neutrophil / lymphocyte; Neutrophil to lymphocyte ratio (NLR) was defined as neutrophil / lymphocyte; Platelet to lymphocyte ratio (PLR) was defined as platelet / lymphocyte; Lymphocyte to monocyte ratio (LMR) was defined as lymphocyte / monocyte.

### Follow-up and outcomes

The survival status of all patients was assessed by outpatient or telephone follow-up. Follow-up was performed every 3–6 months in the first postoperative year, then every 6–12 months for 5 years, and then every year thereafter. The follow-up included physical examination, chest X-ray examination, abdominal and pelvic computed tomography, serum CEA level detection, and colonoscopy. The last follow-up was conducted on July 31, 2022.

The primary outcomes were disease-free survival (DFS) and overall survival (OS). DFS was defined as the time interval from tumor resection to disease recurrence, death, or last follow-up. OS was defined as the time interval from the date of diagnosis to death from any cause or the last follow-up. The secondary outcome was postoperative complications, which were classified according to the modified Clavien–Dindo grading system [19].

### Statistical analysis

According to normality, continuous variables are expressed as mean ± standard deviation or median (interquartile range), and categorical variables are presented as frequencies and percentages. Categorical variables were compared using Pearson’s chi-squared test or Fisher’s exact test. Continuous variables were compared using t-tests or non-parametric tests. The standardized log-rank statistic was used to determine the cut-off value for PINI. The association between continuous PINI and survival in patients with CRC was assessed using restricted cubic splines (RCS). The Kaplan–Meier method was used to plot survival curves, and survival differences were compared using the log-rank test. To solve the multicollinearity problem in multiple linear regression and enhance the stability of the model, the least absolute shrinkage and selection operator (LASSO) logistic regression algorithm was used for feature screening. The Cox proportional hazards model was used to evaluate independent prognostic predictors in patients with CRC. The R package “survival” was used to construct the prognostic nomograms to predict 1–5-year DFS and OS in the patients. The concordance index (C-index) and calibration curve were used to assess the prognostic accuracy of the nomograms. Logistic regression analysis was used to identify independent predictors of postoperative complications in patients with CRC. Finally, we randomly divided the total population into two internal validation datasets at a ratio of 7:3 to evaluate the utility of the nomograms. Statistical significance was set at *p* < 0.05. All statistical analyses were conducted using the R software (version 4.0.2).

## Results

### Clinicopathological characteristics

In total, 1,304 patients with stage I–III CRC were enrolled in this study. The median follow-up period was 67.37 (55.88, 79.86) months. The clinicopathological characteristics are presented in Table 1. Of the patients, 821 (63.0%) were men, and 483 (37.0%) were women, with a mean age of 58.31 (± 13.15) years. Regarding the type of cancer, 685 (52.5%) patients had rectal cancer, and 619 (47.5%) patients had colon cancer. Regarding the cancer stage, 284 (21.8%) patients had stage I, 481 (36.9%) patients had stage II, and 539 (41.3%) patients had stage III cancer. In Chinese patients with CRC, we determined an optimal cut-off value for PINI of 2.85 (Figure S1). Accordingly, 334 (25.6%) patients were identified as having low PINI and 970 (74.4%) patients as having high PINI. A low PINI was closely related to advanced age, low BMI, advanced T stage, colon cancer, large tumor, and high CEA levels. Additionally, patients with a low PINI had a longer hospitalization by approximately 3 days, higher hospital costs, and a higher risk of death than those with a high PINI.

**Table 1:** The relationships between the PINI and clinicopathological features of patients with colorectal cancer

Features	Overall (*n* = 1304)	PINI		*P* value
		Low (*n* = 334)	High (*n* = 970)	
Sex (Man)	821 (63.0)	236 (70.7)	585 (60.3)	0.001
Age (mean (SD))	58.31 (13.00)	61.26 (13.53)	57.30 (12.66)	< 0.001
Age (≥ 60)	655 (50.2)	205 (61.4)	450 (46.4)	< 0.001
BMI (median [IQR])	22.07 (20.00, 24.46)	21.50 (19.15, 23.42)	22.43 (20.21, 24.85)	< 0.001
BMI				< 0.001
Low (< 18.5)	161 (12.3)	64 (19.2)	97 (10.0)	
Normal (18.5–24.9)	762 (58.4)	204 (61.1)	558 (57.5)	
High (≥ 25)	381 (29.2)	66 (19.8)	315 (32.5)	
Hypertension (Yes)	218 (16.7)	61 (18.3)	157 (16.2)	0.428
Diabetes (Yes)	82 (6.3)	23 (6.9)	59 (6.1)	0.696
T stage				0.047
T1	364 (27.9)	88 (26.3)	276 (28.5)	
T2	690 (52.9)	181 (54.2)	509 (52.5)	
T3	250 (19.2)	65 (19.5)	185 (19.1)	
N stage				0.246
N0	765 (58.7)	204 (61.1)	561 (57.8)	
N1	351 (26.9)	91 (27.2)	260 (26.8)	
N2	188 (14.4)	39 (11.7)	149 (15.4)	
TNM stage				0.644
Stage I	284 (21.8)	74 (22.2)	210 (21.6)	
Stage II	481 (36.9)	129 (38.6)	352 (36.3)	
Stage III	539 (41.3)	131 (39.2)	408 (42.1)	
Perineural invasion (Yes)	125 (9.6)	30 (9.0)	95 (9.8)	0.744
Vascular invasion (Yes)	210 (16.1)	46 (13.8)	164 (16.9)	0.208
Macroscopic type				0.011
Protrude type	376 (28.8)	112 (33.5)	264 (27.2)	
Infiltrating type	99 (7.6)	32 (9.6)	67 (6.9)	
Ulcerative type	829 (63.6)	190 (56.9)	639 (65.9)	
Differentiation (Poor)	157 (12.0)	44 (13.2)	113 (11.6)	0.522
Tumor location (Rectal)	685 (52.5)	124 (37.1)	561 (57.8)	< 0.001
Tumor size (median [IQR])	4.50 (3.50, 6.00)	5.50 (4.00, 7.50)	4.00 (3.00, 5.00)	< 0.001
CEA (High)	497 (38.1)	161 (48.2)	336 (34.6)	< 0.001
Surgical method (Endoscopic)	796 (61.0)	155 (46.4)	641 (66.1)	< 0.001
Operation time (median [IQR])	186.00 (148.00, 242.50)	196.50 (163.25, 261.50)	182.00 (143.00, 236.50)	< 0.001
Intraoperative blood loss (median [IQR])	100.00 (50.00, 200.00)	100.00 (80.00, 200.00)	100.00 (50.00, 200.00)	< 0.001
Radiotherapy (Yes)	124 (9.5)	17 (5.1)	107 (11.0)	0.002
Chemotherapy (Yes)	581 (44.6)	138 (41.3)	443 (45.7)	0.183
Death (Yes)	457 (35.0)	168 (50.3)	289 (29.8)	< 0.001
Hospital stays (median [IQR])	17.00 (11.00, 21.00)	19.00 (15.00, 23.75)	16.00 (10.00, 20.00)	< 0.001
Hospitalization cost (median [IQR])	49239.24 (44407.25, 55607.16)	51692.23 (45199.72, 59809.63)	48729.73 (44257.75, 54564.84)	< 0.001

*CRC* Colorectal cancer, *BMI* Body mass index, *PINI* Prognostic immune and nutritional index

### Association of PINI with DFS

**Fig. 1:**
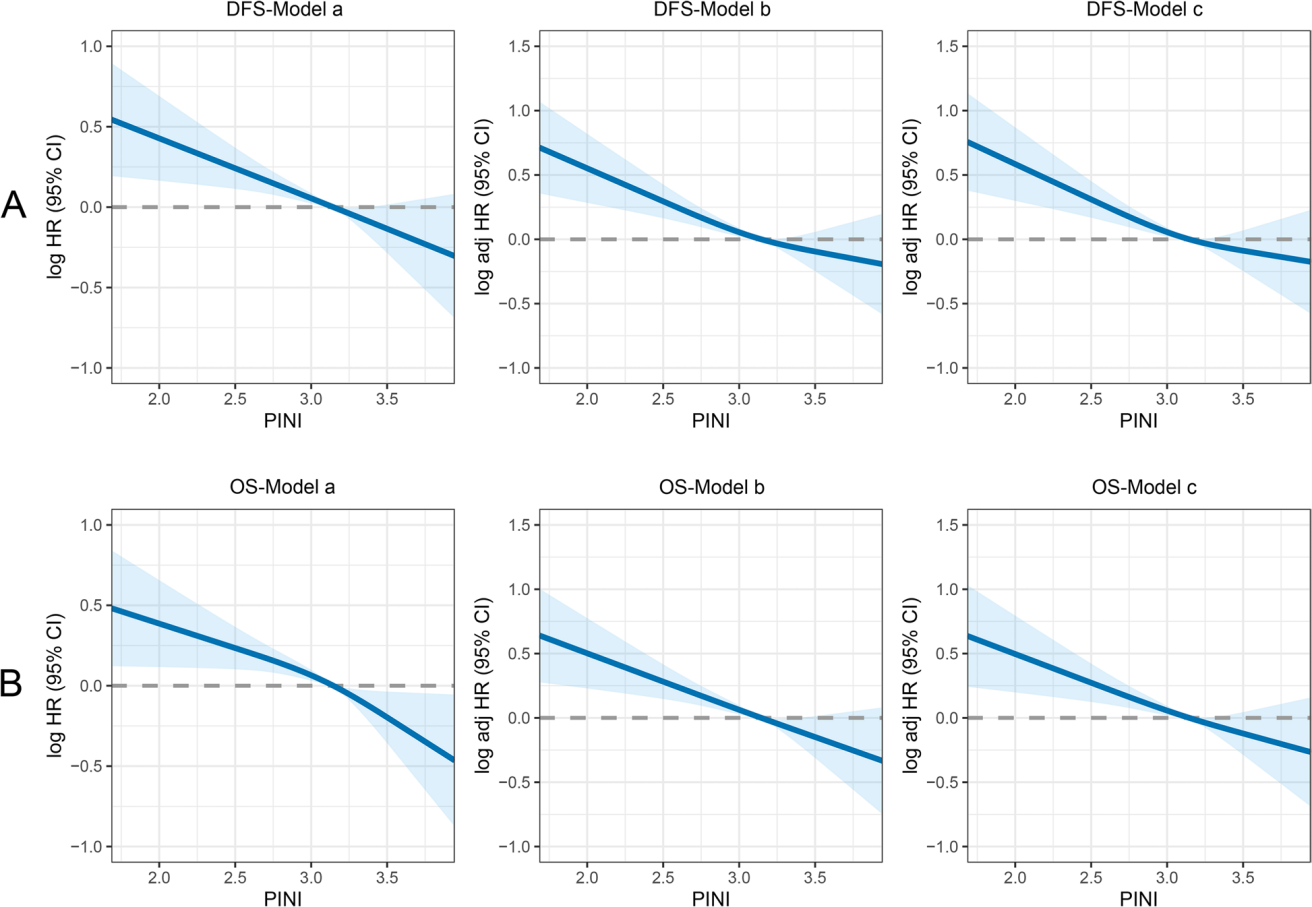
The association between PINI and survival in patients with colorectal cancer. Notes: Model a: No adjusted. Model b: Adjusted for sex, age, and BMI. Model c: Adjusted for sex, age, BMI, hypertension, diabetes, T stage, N stage, metastasis, tumor location, tumor size, perineural invasion, vascular invasion, macroscopic type, differentiation, surgical approach

**Fig. 2:**
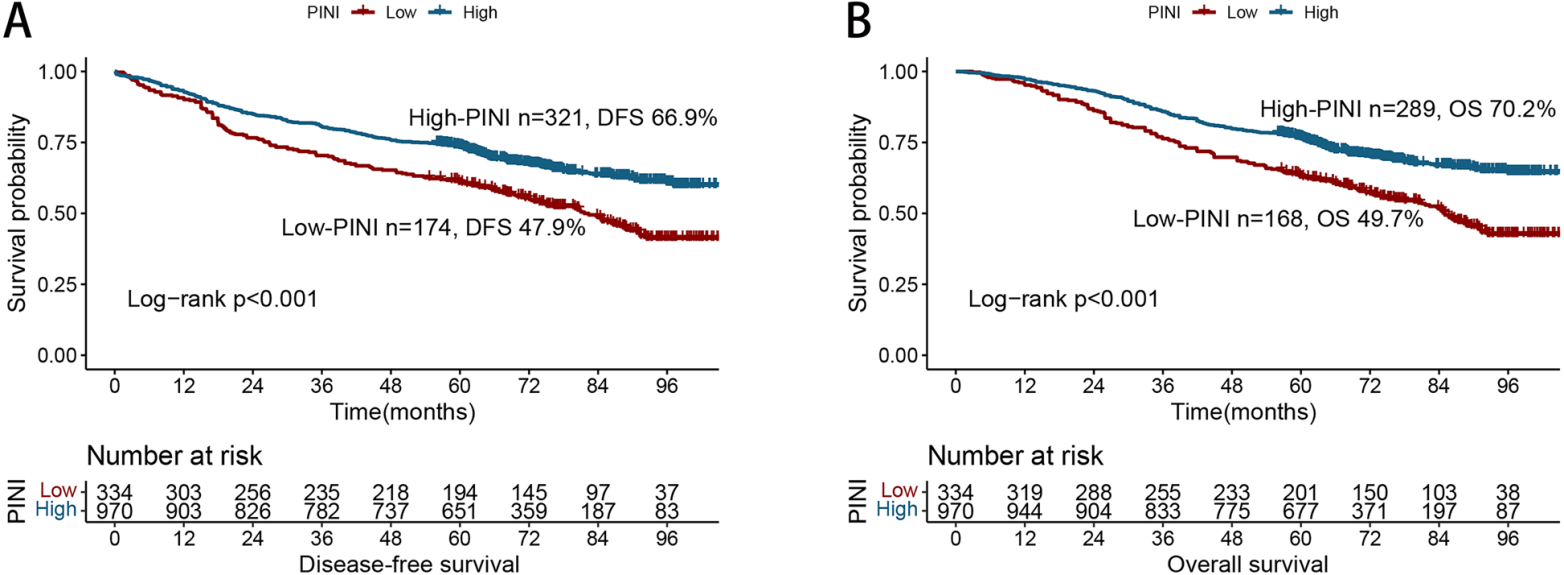
Kaplan-Meier curve of PINI in patients with colorectal cancer. Notes: **A**, Disease-free survival; **B**, Overall survival

An evident positive dose–response relationship between PINI and DFS was observed in the patients with CRC. Specifically, the DFS of the patients with CRC gradually improved with an increase in PINI (Fig. 1A). Compared with patients with a high PINI, those with a low PINI had worse DFS (5-year DFS, 47.9% vs. 66.9%, *p* < 0.001) (Fig. 2A). The PFS of patients with a low PINI was significantly lower than that of patients with a high PINI in both the normal and high CEA groups (Figure S2A, C). PINI was still effective in the prognostic stratification of patients with CRC who had different pathological stages (Figure S3A–C). The multivariate Cox proportional hazards model indicated that PINI was an independent factor affecting DFS in patients with CRC (HR, 0.823; 95% CI, 0.754–0.898; *p* < 0.001) (Table 2). Further subgroup analyses revealed that PINI was an independent factor affecting the prognosis of most subgroups (Figure S4A), further demonstrating the utility of PINI.

**Table 2:** Association between PINI and disease-free survival of patients with colorectal cancer

PINI	Model a	*P* value	Model b	*P* value	Model c	*P* value
Continuous (per SD)	0.845 (0.784,0.912)	< 0.001	0.827 (0.763,0.897)	< 0.001	0.823 (0.754,0.898)	< 0.001
Cutoff value		< 0.001		< 0.001		< 0.001
C1 (~ 2.85)	ref		ref		ref	
C2 (2.85~)	0.618 (0.513,0.743)		0.605 (0.500,0.733)		0.587 (0.477,0.722)	
Quartiles						
Q1 (~ 2.84)	ref		ref		ref	
Q2 (2.84 ~ 3.15)	0.664 (0.526,0.84)	0.001	0.632 (0.498,0.802)	< 0.001	0.619 (0.484,0.793)	< 0.001
Q3 (3.15 ~ 3.38)	0.617 (0.484,0.786)	< 0.001	0.639 (0.498,0.819)	< 0.001	0.603 (0.464,0.783)	< 0.001
Q4 (3.38~)	0.582 (0.454,0.746)	< 0.001	0.558 (0.432,0.719)	< 0.001	0.542 (0.412,0.711)	< 0.001
p for trend		< 0.001		< 0.001		< 0.001

Model a: No adjusted

Model b: Adjusted for sex, age, T stage and N stage

Model c: Adjusted for sex, age, BMI, hypertension, diabetes, T stage, N stage, tumor location, tumor size, perineural invasion, vascular invasion, macroscopic type, differentiation, surgical approach, CEA level, radiotherapy, and chemotherapy

### Association of PINI with OS

The multivariate-adjusted RCS revealed a significant positive relationship between continuous PINI and OS in patients with CRC (Fig. 1B). The Kaplan–Meier survival curves revealed that the OS of the low PINI group was significantly worse than that of the high PINI group (5-year OS: 49.7% vs. 70.2%, *p* < 0.001) (Fig. 2B). For the early stages (TNM stages I–II), the OS of the patients with a high PINI was significantly poorer than that of patients with a low PINI (Figure S3D, E). For the advanced stage (TNM III stage), PINI still provided effective prognostic differentiation of patients with CRC (Figure S3F). PINI also effectively differentiated the OS of patients with CRC in the different CEA subgroups (Figure S2B, D). After adjusting for confounders, PINI was independently associated with OS in the patients with CRC (HR, 0.833; 95% CI, 0.761–0.912; *p* < 0.001). Thus, whether as a continuous or categorical variable, PINI was independently associated with the prognosis of patients with CRC (Table 3). Additionally, PINI was an independent factor affecting OS in most subgroups of patients with CRC (Figure S4B).

**Table 3:** Association between PINI and overall survival of patients with colorectal cancer

PINI	Model a	*P* value	Model b	*P* value	Model c	*P* value
Continuous (per SD)	0.839 (0.777,0.906)	< 0.001	0.824 (0.759,0.895)	< 0.001	0.833 (0.761,0.912)	< 0.001
Cutoff value		< 0.001		< 0.001		< 0.001
C1 (~ 2.85)	ref		ref		ref	
C2 (2.85~)	0.574 (0.474,0.694)		0.56 (0.459,0.682)		0.561 (0.453,0.696)	
Quartiles						
Q1 (~ 2.84)	ref		ref		ref	
Q2 (2.84 ~ 3.15)	0.632 (0.496,0.805)	< 0.001	0.592 (0.463,0.757)	< 0.001	0.592 (0.458,0.765)	< 0.001
Q3 (3.15 ~ 3.38)	0.558 (0.432,0.72)	< 0.001	0.584 (0.45,0.759)	< 0.001	0.575 (0.436,0.757)	< 0.001
Q4 (3.38~)	0.547 (0.422,0.708)	< 0.001	0.523 (0.402,0.682)	< 0.001	0.534 (0.402,0.708)	< 0.001
p for trend		< 0.001		< 0.001		< 0.001

Model a: No adjusted

Model b: Adjusted for sex, age, T stage and N stage

Model c: Adjusted for sex, age, BMI, hypertension, diabetes, T stage, N stage, tumor location, tumor size, perineural invasion, vascular invasion, macroscopic type, differentiation, surgical approach, CEA level, radiotherapy, and chemotherapy

### Association of PINI with postoperative complications

In this study, postoperative complications occurred in 269 patients (20.6%), including 20 cases of intestinal obstruction, 33 cases of anastomotic problems, 72 cases of wound problems, 37 cases of pulmonary infection, 20 cases of gastrointestinal problems, 5 cases of abdominal infection, 82 cases of other mild complications. According to the modified Clavien–Dindo complication classification system, 135 (10.4%) cases were grade I complications, 98 (7.5%) cases were grade II complications, 16 (1.2%) cases were grade IIIa complications, 10 (0.8%) cases were grade IIIb complications, five (0.4%) cases were grade IVa complications, four (0.3%) cases were grade IVb complications, and one (0.1%) case was a grade V complication. The frequency of postoperative complications in the low PINI group was higher than that in the high PINI group (31.1% vs. 17.0%; *p* < 0.001). Furthermore, in the subgroup analysis, the postoperative complication rates of grade II (*p* < 0.001) and grade IIIa (*p* < 0.001) were significantly higher in the low PINI group (Table S1). In the univariate logistic regression analysis, PINI was significantly associated with postoperative complications (odds ratio [OR], 0.682; 95% confidence interval [CI], 0.603–0.773; *p* < 0.001). After adjusting for confounding variables, the risk of postoperative complications in patients with CRC was reduced by approximately 29% (OR: 0.710, 95%CI: 0.610–0.810, *p* < 0.001) for each additional SD of PINI (Table S2).

### Comparison of PINI with other prognostic indicators

**Fig. 3:**
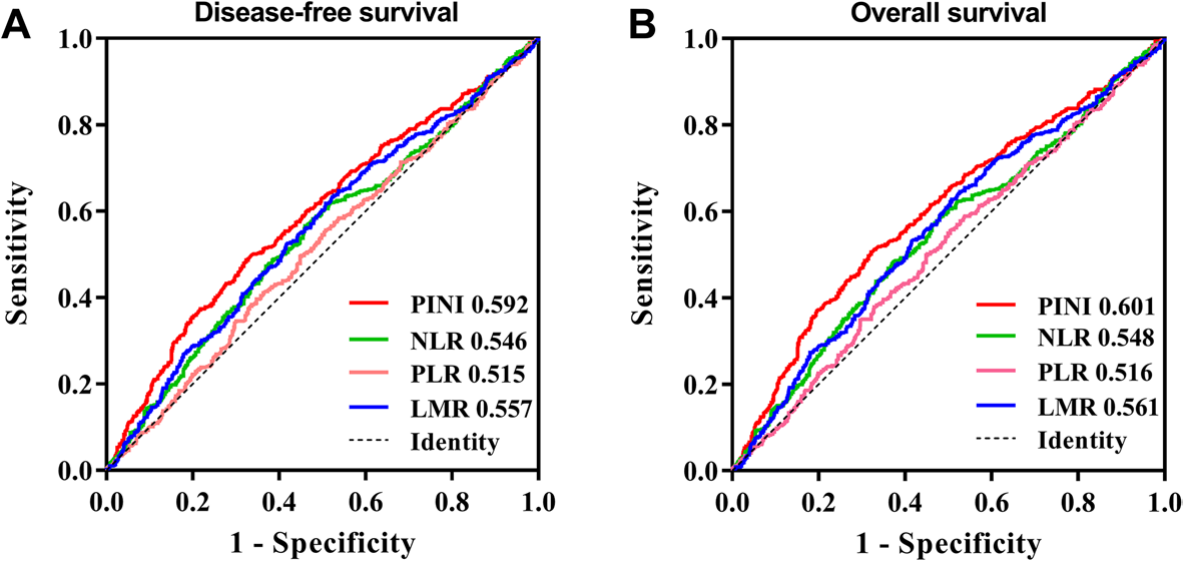
The ROC curves of prognostic indicators for the prediction of prognosis. Notes: **A**, Disease-free survival; **B**, Overall survival

We compared the ability of other prognostic indicators to predict clinical outcomes in CRC patients through ROC curves (Fig. 3). For DFS, the area under the curve (AUC) of PINI was (0.592, 95% CI: 0.560–0.624, *p* < 0.001), which was higher than that of NLR (0.546, 95% CI: 0.513–0.578, *p* = 0.006), PLR (0.515, 95% CI: 0.483–0.548, *p* = 0.356), and LMR (0.557, 95% CI: 0.525–0.589, *p* < 0.001). For OS, the AUC of PINI (0.601, 95% CI: 0.568–0.633, *p* < 0.001) is also better than that of NLR (0.548, 95% CI: 0.515–0.582, *p* = 0.004), PLR (0.516, 95% CI: 0.483–0.549, *p* = 0.345) and LMR (0.561, 95% CI: 0.529–0.594, *p* < 0.001).

### Feature selection using the LASSO logistic regression algorithm

**Fig. 4:**
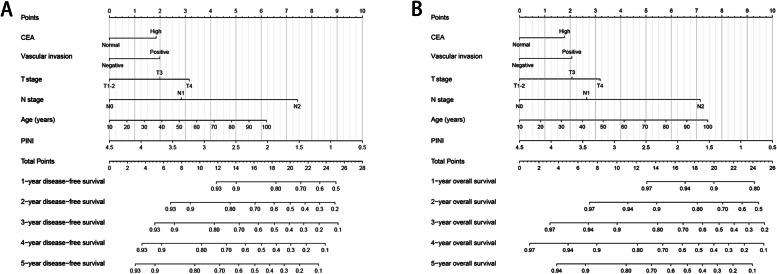
Construction the PINI-based prognostic nomograms in patients with colorectal cancer. Notes: **A**, The disease-free survival nomogram; **B**, The overall survival nomogram

Next, we screened for the most effective prognostic features of patients with CRC using the LASSO logistic regression algorithm. When the optimal lambda values of DFS and OS were 0.054 and 0.056, respectively (Figure S5A, B), six features with non-zero coefficients were identified as the optimal prognostic features, namely, age, T stage, N stage, vascular invasion, serum CEA level, and PINI. The six features were included in the multivariate Cox proportional hazards model, and the results revealed that all six features were independent factors affecting DFS/OS in patients with CRC (Tables S3 and S4). Furthermore, we developed prognostic nomograms based on these six features to predict the 1–5-year DFS/OS (Fig. 4A, B). The nomograms revealed that with the increase in CEA, appearance of vascular invasion, progression of T stage/N stage, increase in age, and decrease in PINI, the prediction score increased, indicating that the risk of poor prognosis also increased.

### Evaluation and internal validation of survival nomograms

The C-indices for DFS and OS nomograms were 0.688 (95% CI: 0.664–0.712) and 0.696 (95% CI: 0.672–0.721), respectively. The calibration curves of the 3- and 5-year DFS (Figure S6A, B) and OS (Figure S6C, D) demonstrated the best agreement between the predicted survival probabilities and actual observations. Thus, prognostic nomograms may accurately predict the prognosis of patients with CRC. Subsequently, we performed a randomized internal validation to verify the utility of these nomograms. According to the ratio of 7:3, the total population was divided into validation (916) and validation (388) groups (Table S5). In validation a, the C-indices of DFS and OS were 0.683 (95% CI: 0.655–0.711) and 0.691 (95% CI: 0.662–0.720), respectively, whereas in validation b, the C-indices of DFS and OS were 0.714 (95% CI: 0.674–0.754) and 0.723 (95% CI: 0.680–0.766), respectively. The calibration curves for both 3- and 5-year PFS and OS demonstrated the best agreement between the predicted survival probabilities and actual observations in both validation a (Figure S7A) and validation b (Figure S7B).

## Discussion

In this study, we investigated the clinical significance of PINI in patients with stage I–III CRC who underwent primary tumor resection. PINI was identified as a strong predictor of postoperative complications, recurrence, and poor prognosis in patients with CRC. PINI has also been shown to be superior to other prognostic indicators such as NLR, PLR, and LMR in predicting the prognosis of CRC patients. Furthermore, we screened out the optimal prognostic features, including PINI, using the LASSO logistic regression algorithm to construct prognostic prediction models for patients with CRC. The results of the C-index and calibration curve analysis confirmed the good predictive performance of these PINI-based nomograms. These analyses provide a useful reference for the application of PINI in clinical practice.

PINI is an emerging prognostic indicator, and its cut-off value for clinical application in the Chinese population with CRC is unclear. Based on survival status, we determined that the optimal cut-off value of PINI was 2.85 for the Chinese population with CRC. Based on this cut-off value, PINI was able to effectively stratify DFS or OS in such patients. The risk of poor prognosis in the low PINI group was 1.7 times higher than that in the high PINI group. Additionally, PINI was demonstrated to be independently associated with DFS and OS in patients with CRC, and this association was observed in most subgroups. Systemic inflammation and malnutrition are important factors that lead to tumor recurrence and poor outcomes [7, 20, 21]. Here, a low PINI was strongly associated with a poor tumor phenotype (advanced T stage, large tumors, and high CEA levels) and poorer physical status (older age and low BMI). Thus, a low PINI may represent an increase in systemic inflammation and a decrease in nutritional status, which may predict worse clinical outcomes. Pathological stage remains a key factor affecting the prognosis of patients with CRC. PINI serves as a useful adjunct to achieve more accurate prognostic stratification for patients with the same pathological stage. The combined evaluation of pathological stage and PINI may be a valuable means of predicting postoperative recurrence and prognosis, which will provide favorable evidence for improving clinical decision-making.

In this study, PINI was identified as an effective predictor of postoperative complications in patients with CRC. Patients with a low PINI had a higher risk of postoperative complications, which was approximately twice as high as those with a high PINI. Studies have demonstrated that postoperative complications are closely associated with systemic inflammation and malnutrition [22–24]. PINI integrates parameters that reflect the nutritional status (serum albumin) and systemic inflammation (monocyte), thus giving it a natural advantage in predicting postoperative complications in patients with CRC. In this study, we also compare the value of PINI and conventional prognostic indicators in predicting the prognosis of patients with CRC. We find that PINI is better than conventional prognostic indicators. This may be because the PINI is effectively combined with albumin and monocyte according to the coefficient, which is superior to conventional combination of multiplication and division. Based on the above analysis, PINI integrates the advantages of systemic inflammation and nutrition-related biomarkers and can be used to evaluate the disease burden and predict poor prognosis in patients with CRC.

From a large number of clinical features, we then screened for six features that are the most associated with the prognosis of CRC using the LASSO logistic regression algorithm. To facilitate intuitive use in clinical work, we constructed prognostic nomograms with these features. These nomograms, which integrate the advantages of personal conditions, tumor characteristics, serum tumor markers, and nutritional inflammation-related markers, can be used for personalized assessment of 1–5-year PFS and OS in patients with CRC. We subsequently confirmed the good predictive accuracy of these nomograms through randomized internal validation. These prognostic nomograms can provide a quantitative reference for the prognostic evaluation of patients with stage I–III CRC so that appropriate treatment strategies can be formulated for such patients more conveniently and individually.

To the best of our knowledge, this study is the first to report PINI as a strong predictor of postoperative complications, DFS, and OS in Chinese patients with stage I–III CRC. Additionally, we constructed PINI-based nomograms. These analyses provide a positive contribution to the implementation of tertiary prevention of CRC.

However, this study had some limitations. First, this study was retrospective in nature, so bias in patient selection and study design may be inherent. Second, although internal validation was performed, the prognostic nomograms should be externally validated more extensively before clinical application. Last, because this study only collected PINI data from a single episode, the impact of PINI trajectory changes on CRC prognosis could not be explored.

## Conclusion

PINI is an effective biomarker for predicting postoperative complications, DFS, and OS in patients with stage I–III CRC. PINI-based nomograms can provide a personalized reference for prognostic evaluation and clinical decision-making for patients with CRC.

## Supplementary Information

**Additional file 1: Figure S1.** The cur-point of PINI in CRC patients. **Figure S2.** Stratified survival analysis of PINI based on different CEA level. **Figure S3.** Stratified survival analysis of PINI based on different pathological stages. **Figure S4.** The association between PINI and hazard risk of survival in various subgroups. (A, Disease-free survival, B, Overall survival). **Figure S5.** Feature selection using least absolute shrinkage and selection operator (LASSO) logistic regression. **Figure S6.** Calibration curve of the disease-free survival and overall survival nomograms. **Figure S7.** Calibration curve at randomize internal validation cohorts. **Table S1.** Details of postoperative complications according to modified Clavien grading system. **Table S2.** Univariate and multivariate Logistic regression analysis of complications in CRC patients. **Table S3.** The Cox regression analysis of clinicopathological features screened by LASSO regression on disease-free survival. **Table S4.** The Cox regression analysis of clinicopathological features screened by LASSO regression on disease-free survival. **Table S5.** The clinicopathological Features of two validation cohorts in CRC patients. 

## Data Availability

All data needed to evaluate the conclusions of the study are presented in this paper and/or the Supplementary Materials. Additional data related to this study is available upon request to authors/corresponding author.
