# Supplementary material for: Prognostic significance of preoperative prognostic immune and nutritional index in patients with stage I–III colorectal cancer
Source: BMC Cancer. 2022 Dec 16;22:1316. doi: 10.1186/s12885-022-10405-w (PMC9756500; doi:10.1186/s12885-022-10405-w)
Supplement: Supplementary file 1 — Additional file 1: Figure S1. The cur-point of PINI in CRC patients. Figure S2. Stratified survival analysis of PINI based on different CEA level. Figure S3. Stratified survival analysis of PINI based on different pathological stages. Figure S4. The association between PINI and hazard risk of survival in various subgroups. (A, Disease-free survival, B, Overall survival). Figure S5. Feature selection using least absolute shrinkage and selection operator (LASSO) logistic regression. Figure S6. Calibration curve of the disease-free survival and overall survival nomograms. Figure S7. Calibration curve at randomize internal validation cohorts. Table S1. Details of postoperative complications according to modified Clavien grading system. Table S2. Univariate and multivariate Logistic regression analysis of complications in CRC patients. Table S3. The Cox regression analysis of clinicopathological features screened by LASSO regression on disease-free survival. Table S4. The Cox regression analysis of clinicopathological features screened by LASSO regression on disease-free survival. Table S5. The clinicopathological Features of two validation cohorts in CRC patients. [file 12885_2022_10405_MOESM1_ESM.docx]

**Figure S1.** The cur-point of PINI in CRC patients.

**
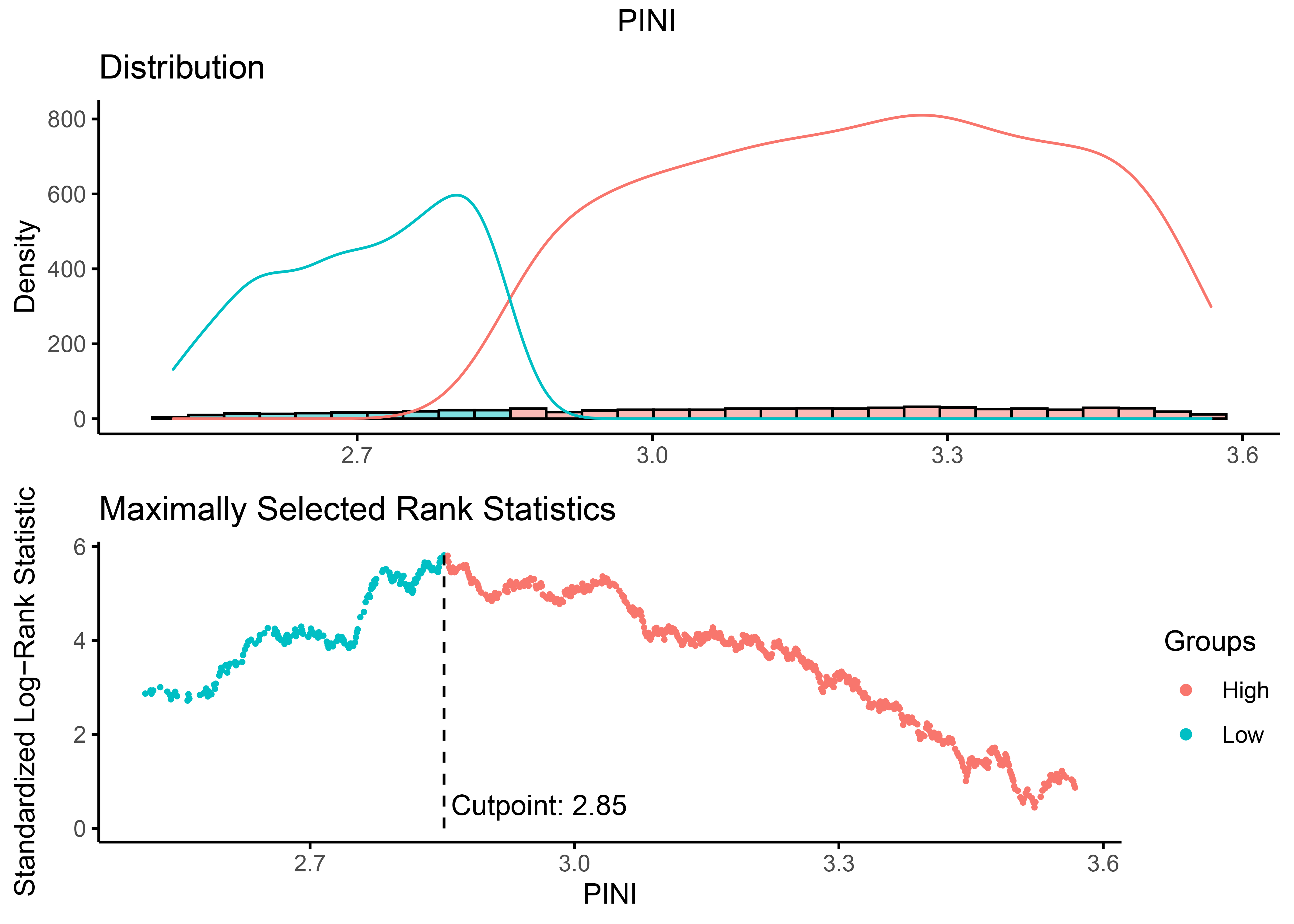
**

**Figure S2.** Stratified survival analysis of PINI based on different CEA level.


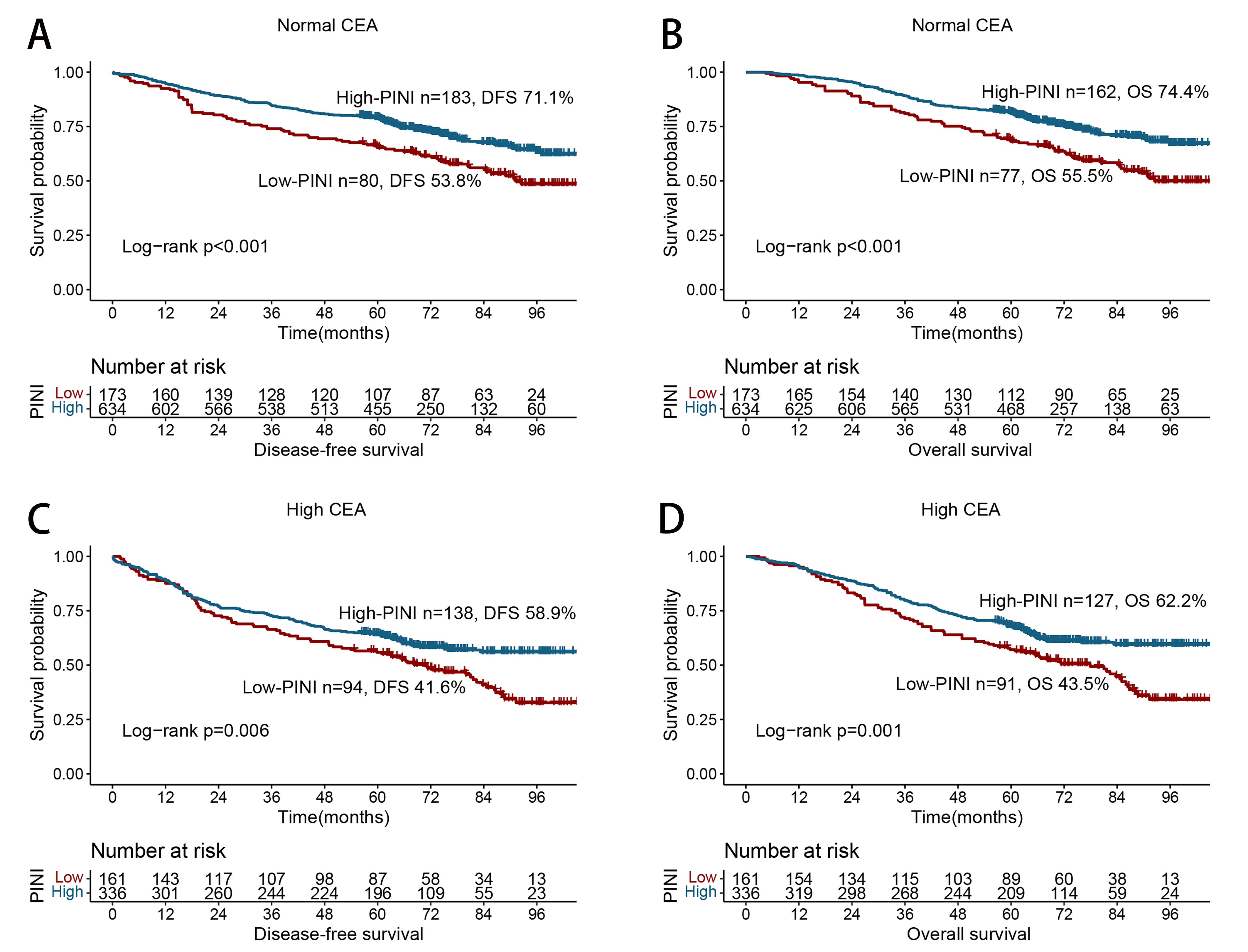


Notes: A, Disease-free survival of PINI at normal CEA level; B, Overall survival of PINI at normal CEA level; C, Disease-free survival of PINI at high CEA level; D, Overall survival of PINI at high CEA level.

**Figure S3.** Stratified survival analysis of PINI based on different pathological stages.


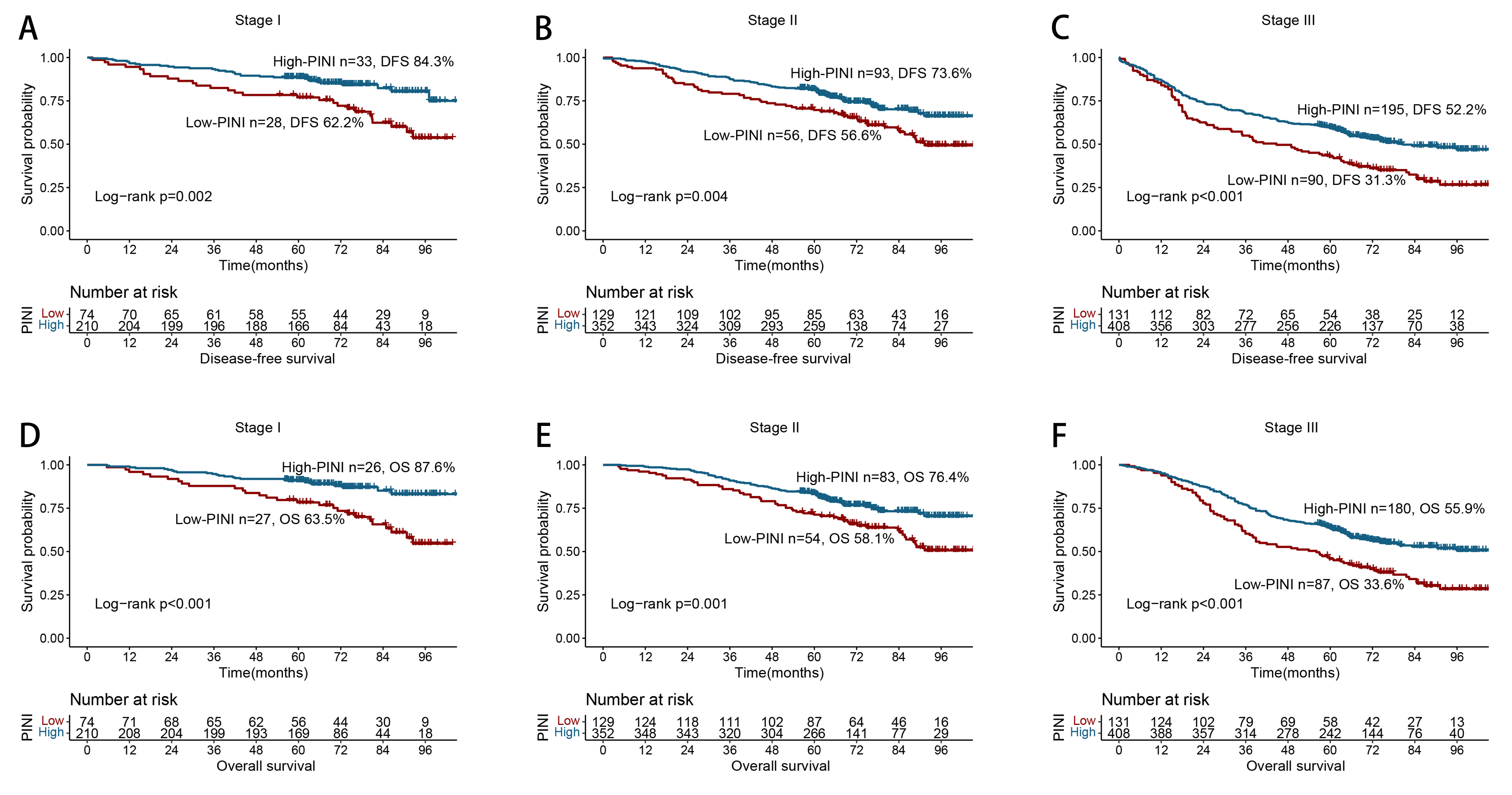


Notes: A, Disease-free survival of stage I; B, Disease-free survival of stage II; C, Disease-free survival of stage III; D, Overall survival of stage I; E, Overall survival of stage II; F, Overall survival of stage III.

**Figure S4.** The association between PINI and hazard risk of survival in various subgroups. (A, Disease-free survival, B, Overall survival).


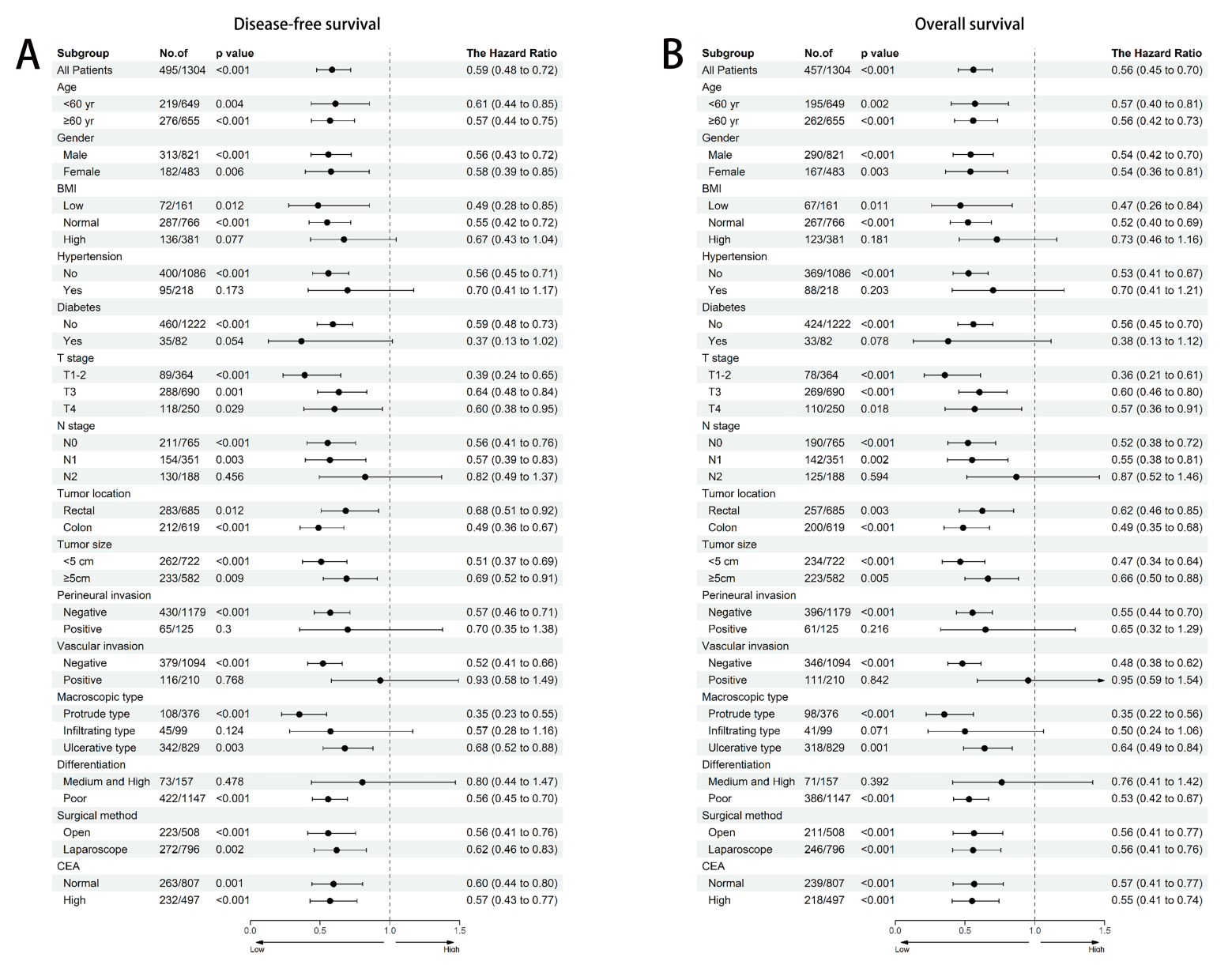


**Figure S5.** Feature selection using least absolute shrinkage and selection operator (LASSO) logistic regression.

**
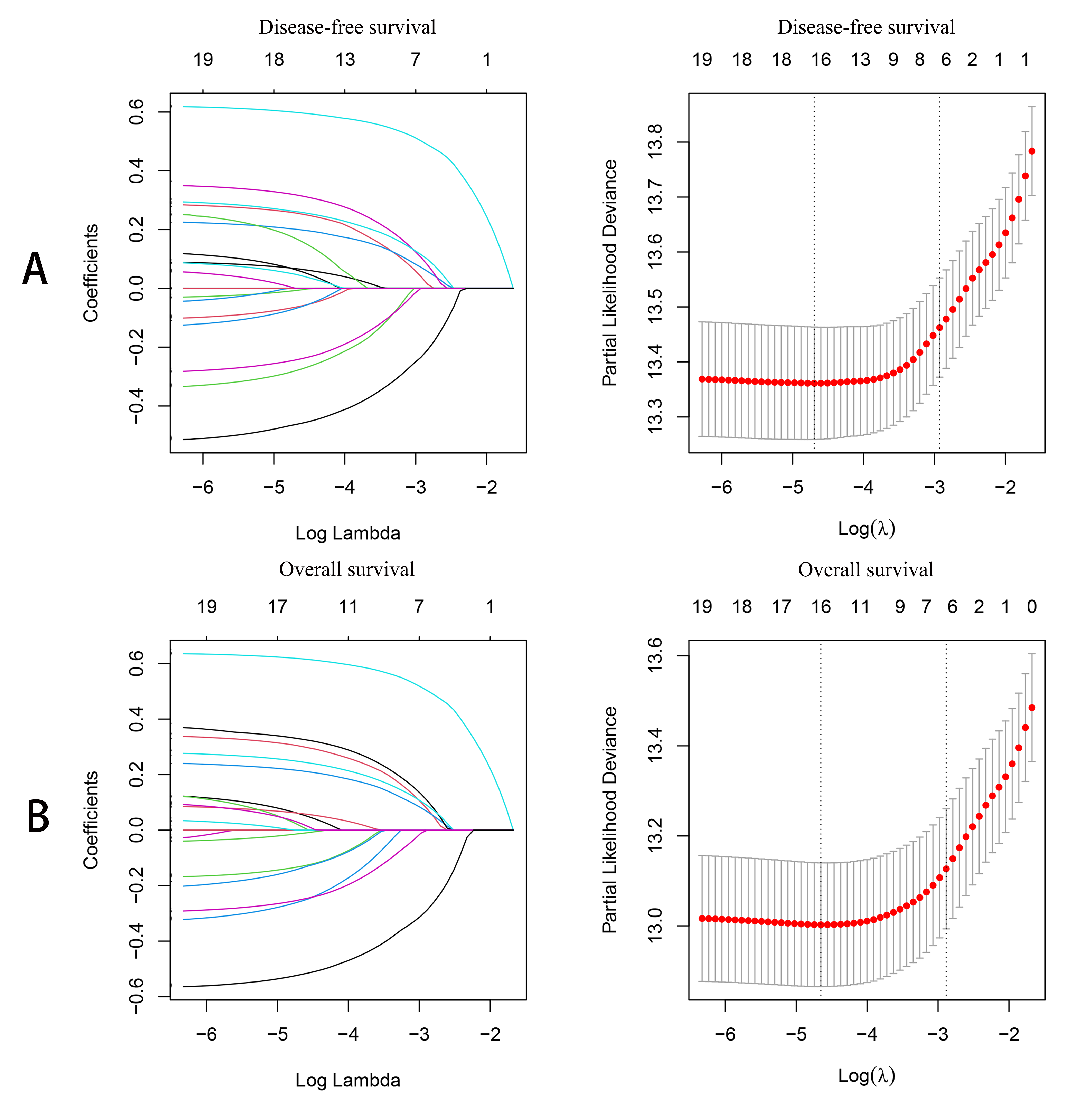
**

Notes: A, Disease-free survival; B, Overall survival.

**Figure S6.** Calibration curve of the disease-free survival and overall survival nomograms.

**
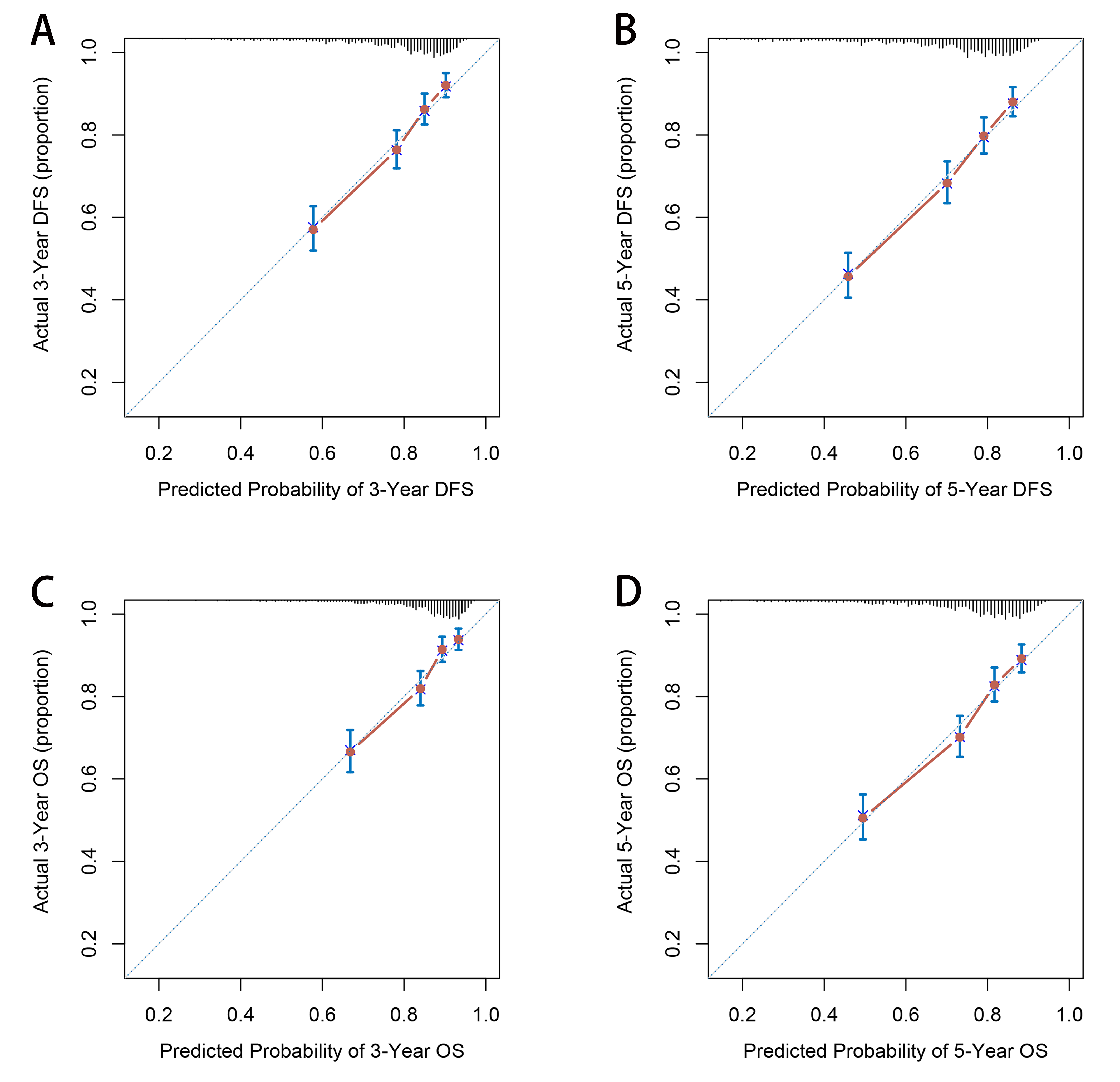
**

Notes: A, 3-year disease-free survival; B, 5-year disease-free survival; C, 3-year overall survival; D, 5-year overall survival.

**Figure S7.** Calibration curve at randomize internal validation cohorts.

**
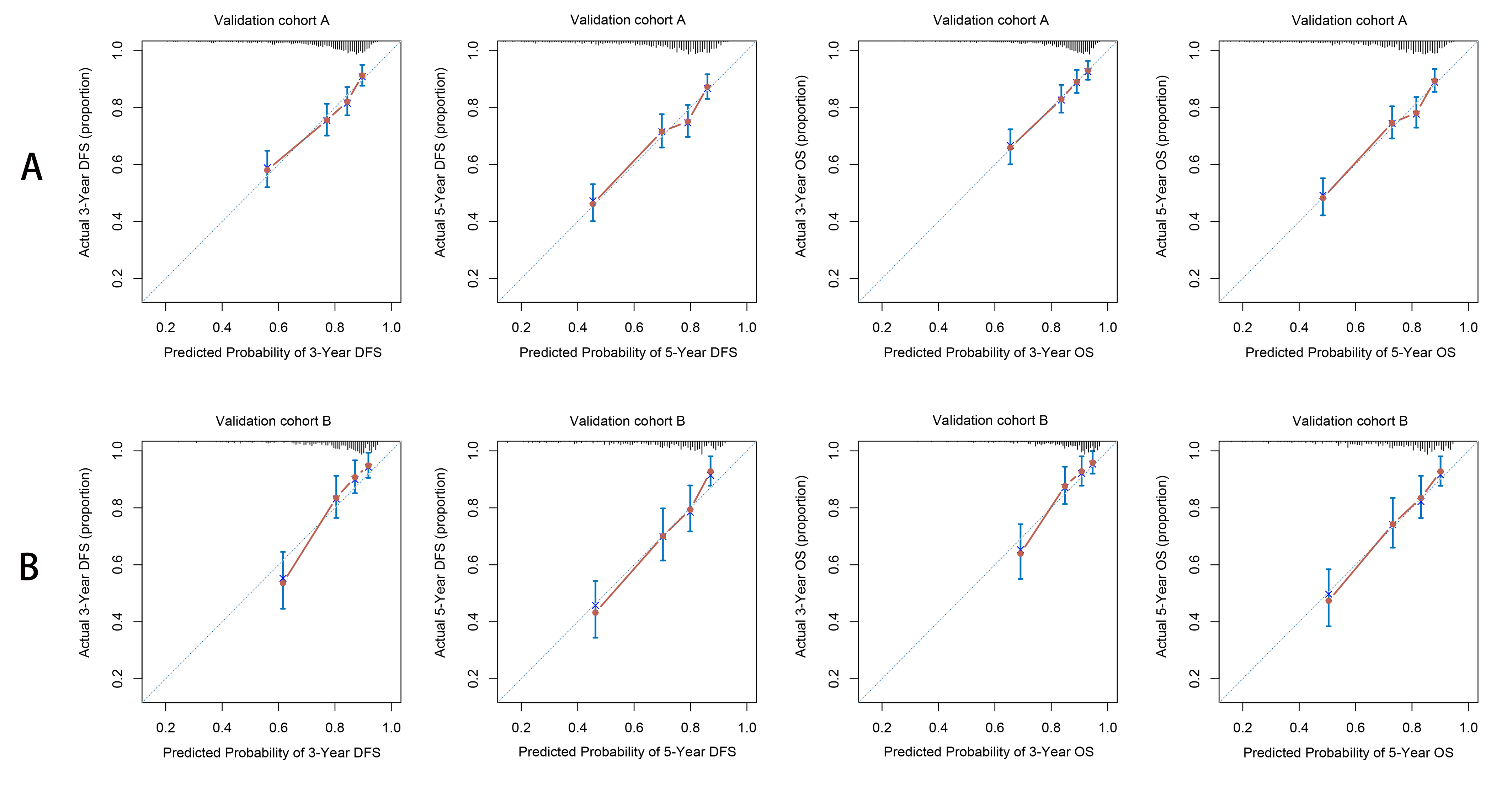
**

**Notes:** A, Validation a cohort; B, Validation b cohort.

**Table S1** Details of postoperative complications according to modified Clavien grading system.

| Grade | Total (n=1304) | PINI | | p value |
| --- | --- | --- | --- | --- |
|  |  | Low (n = 334) | High (n = 970) |  |
| Total complications | 269 (20.6) | 104 (31.1) | 165 (17.0) | <0.001 |
| Grade I | 135 (10.4) | 42 (12.6) | 93 ( 9.6) | 0.122 |
| Grade II | 98 ( 7.5) | 43 (12.9) | 55 ( 5.7) | <0.001 |
| Grade III |  |  |  |  |
| Grade IIIa | 16 ( 1.2) | 9 ( 2.7) | 7 ( 0.7) | 0.005 |
| Grade IIIb | 10 ( 0.8) | 4 ( 1.2) | 6 ( 0.6) | 0.289 |
| Grade IV |  |  |  |  |
| Grade IVa | 5 ( 0.4) | 3 ( 0.9) | 2 ( 0.2) | 0.110 |
| Grade IVb | 4 ( 0.3) | 2 ( 0.6) | 2 ( 0.2) | 0.272 |
| Grade V | 1 ( 0.1) | 1 ( 0.3) | 0 ( 0.0) | 0.474 |

**Table S2.** Univariate and multivariate Logistic regression analysis of complications in CRC patients.

| Complication | | | | | | |
| --- | --- | --- | --- | --- | --- | --- |
| NAR | Model a | p value | Model b | p value | Model b | p value |
| Continuous (per SD) | 0.682 (0.603,0.773) | <0.001 | 0.688 (0.605,0.783) | <0.001 | 0.710 (0.610,0.810) | <0.001 |
| Cutoff value |  |  |  |  |  |  |
| C1 (~2.85) | ref |  | ref |  | ref |  |
| C2 (2.85~) | 0.453 (0.341,0.603) | <0.001 | 0.467 (0.347,0.628) | <0.001 | 0.501 (0.364,0.688) | <0.001 |
| Quartiles |  |  |  |  |  |  |
| Q1 (~2.84) | ref |  | ref |  | ref |  |
| Q2 (2.84~3.15) | 0.602 (0.423,0.857) | 0.005 | 0.600 (0.419,0.859) | 0.005 | 0.619 (0.426,0.900) | 0.012 |
| Q3 (3.15~3.38) | 0.447 (0.307,0.651) | <0.001 | 0.447 (0.307,0.651) | <0.001 | 0.505 (0.338,0.755) | <0.001 |
| Q4 (3.38~) | 0.348 (0.234,0.516) | <0.001 | 0.558 (0.432,0.719) | <0.001 | 0.382 (0.248,0.589) | <0.001 |
| p for trend |  | <0.001 |  | <0.001 |  | <0.001 |

**Table S3** The Cox regression analysis of clinicopathological features screened by LASSO regression on disease-free survival.

| Characteristic | Disease-free survival | | | |
| --- | --- | --- | --- | --- |
|  | Univariate analysis | | Multivariate analysis | |
|  | HR (95%CI) | P value | HR (95%CI) | P value |
| Age | 1.011 (1.004-1.018) | 0.002 | 1.012 (1.005 - 1.019) | 0.001 |
| PINI | 0.845 (0.784,0.912) | <0.001 | 0.83 (0.765 - 0.9) | <0.001 |
| T stage |  |  |  |  |
| T1 | Ref. |  |  |  |
| T2 | 1.906 (1.502-2.417) | <0.001 | 1.396 (1.09 - 1.787) | 0.008 |
| T3 | 2.436 (1.848-3.211) | <0.001 | 1.701 (1.275 - 2.269) | <0.001 |
| N stage |  | <0.001 |  |  |
| N0 | Ref. |  |  |  |
| N1 | 1.798 (1.461-2.213) | <0.001 | 1.623 (1.31 - 2.009) | <0.001 |
| N2 | 4.024 (3.23-5.014) | <0.001 | 3.534 (2.791 - 4.474) | <0.001 |
| Vascular invasion (Positive) | 1.951 (1.584-2.403) | <0.001 | 1.414 (1.137 - 1.759) | 0.002 |
| CEA (≥5ng/ml) | 1.648 (1.381-1.966) | <0.001 | 1.363 (1.138 - 1.633) | 0.001 |

**Table S4** The Cox regression analysis of clinicopathological features screened by LASSO regression on disease-free survival.

| Characteristic | Overall survival | | | |
| --- | --- | --- | --- | --- |
|  | Univariate analysis | | Multivariate analysis | |
|  | HR (95%CI) | P value | HR (95%CI) | p value |
| Age | 1.014 (1.006-1.021) | <0.001 | 1.015 (1.008 - 1.023) | <0.001 |
| PINI | 0.839 (0.777,0.906) | <0.001 | 0.823 (0.757 - 0.894) | <0.001 |
| T stage |  |  |  |  |
| T1 | Ref. |  |  |  |
| T2 | 1.994 (1.55-2.566) | <0.001 | 1.442 (1.11 - 1.874) | 0.006 |
| T3 | 2.556 (1.91-3.419) | <0.001 | 1.763 (1.301 - 2.389) | <0.001 |
| N stage |  | <0.001 |  |  |
| N0 | Ref. |  |  |  |
| N1 | 1.798 (1.446-2.235) | <0.001 | 1.612 (1.289 - 2.016) | <0.001 |
| N2 | 4.122 (3.285-5.173) | <0.001 | 3.596 (2.817 - 4.589) | <0.001 |
| Vascular invasion (Positive) | 2.022 (1.632-2.505) | <0.001 | 1.459 (1.166 - 1.826) | 0.001 |
| CEA (≥5ng/ml) | 1.684 (1.401-2.023) | <0.001 | 1.368 (1.134 - 1.65) | 0.001 |

**Table S5** The clinicopathological Features of two validation cohorts in CRC patients.

| Features | Validation a  (n = 916) | Validation b  (n = 388) | p value |
| --- | --- | --- | --- |
| Gender(male) | 582 (63.5) | 239 (61.6) | 0.548 |
| Age (mean (SD)) | 58.28 (12.95) | 58.38 (13.12) | 0.903 |
| Age (≥60) | 459 (50.1) | 196 (50.5) | 0.941 |
| BMI (median [IQR]) | 22.00 (19.94, 24.35) | 22.22 (20.20, 24.81) | 0.116 |
| BMI |  |  | 0.593 |
| Low (<18.5) | 115 (12.6) | 46 (11.9) |  |
| Normal (18.5-24.9) | 541 (59.1) | 221 (57.0) |  |
| High (≥25） | 260 (28.4) | 121 (31.2) |  |
| Hypertension (Yes) | 155 (16.9) | 63 (16.2) | 0.825 |
| Diabetes (Yes) | 58 (6.3) | 24 ( 6.2) | 0.997 |
| T stage |  |  |  |
| T1 | 259 (28.3) | 105 (27.1) | 0.847 |
| T2 | 480 (52.4) | 210 (54.1) |  |
| T3 | 177 (19.3) | 73 (18.8) |  |
| N stage |  |  |  |
| N0 | 538 (58.7) | 227 (58.5) | 0.911 |
| N1 | 244 (26.6) | 107 (27.6) |  |
| N2 | 134 (14.6) | 54 (13.9) |  |
| TNM stage |  |  |  |
| Stage I | 200 (21.8) | 84 (21.6) | 0.980 |
| Stage II | 339 (37.0) | 142 (36.6) |  |
| Stage III | 377 (41.2) | 162 (41.8) |  |
| Perineural invasion (Yes) | 91 (9.9) | 34 ( 8.8) | 0.579 |
| Vascular invasion (Yes) | 145 (15.8) | 65 (16.8) | 0.740 |
| Macroscopic type |  |  |  |
| Protrude type | 266 (29.0) | 110 (28.4) | 0.408 |
| Infiltrating type | 75 (8.2) | 24 (6.2) |  |
| Ulcerative type | 575 (62.8) | 254 (65.5) |  |
| Differentiation (Poor) | 105 (11.5) | 52 (13.4) | 0.373 |
| Tumor location (Rectal) | 476 (52.0) | 209 (53.9) | 0.570 |
| Tumor size (median [IQR]) | 4.50 (3.50, 6.00) | 4.50 (3.00, 6.00) | 0.770 |
| CEA (High) | 342 (37.3) | 155 (39.9) | 0.409 |
| Surgical method (Endoscopic) | 570 (62.2) | 226 (58.2) | 0.199 |
| Operation time (median [IQR]) | 185.50 (150.00, 241.25) | 189.00 (144.00, 244.25) | 0.820 |
| Intraoperatve blood loss(median [IQR]) | 100.00 (50.00, 200.00) | 100.00 (50.00, 200.00) | 0.649 |
| Radiotherapy (Yes) | 79 ( 8.6) | 45 (11.6) | 0.116 |
| Chemotherapy (Yes) | 405 (44.2) | 176 (45.4) | 0.749 |
| Death (Yes) | 320 (34.9) | 137 (35.3) | 0.947 |

**Table Note:** CRC, colorectal cancer; BMI, body mass index; PINI, prognostic immune and nutritional index.
